# Transmit Power Optimization for Simultaneous Wireless Information and Power Transfer-Assisted IoT Networks with Integrated Sensing and Communication and Nonlinear Energy Harvesting Model

**DOI:** 10.3390/e27050456

**Published:** 2025-04-24

**Authors:** Chengrui Zhou, Xinru Wang, Yanfei Dou, Xiaomin Chen

**Affiliations:** School of Information Science and Technology, Nantong University, Nantong 226019, China; chengrui@stmail.ntu.edu.cn (C.Z.); xinru@stmail.ntu.edu.cn (X.W.); yanfei@stmail.ntu.edu.cn (Y.D.)

**Keywords:** simultaneous wireless information and power transfer, integrated sensing and communication, semi-definite relaxation

## Abstract

Integrated sensing and communication (ISAC) can improve the energy harvesting (EH) efficiency of simultaneous wireless information and power transfer (SWIPT)-assisted IoT networks by enabling precise energy harvest. However, the transmit power is increased in the hybrid system due to the fact that the sensing signals are required to be transferred in addition to the communication data. This paper aims to tackle this issue by formulating an optimization problem to minimize the transmit power of the base station (BS) under a nonlinear EH model, considering the coexistence of power-splitting users (PSUs) and time-switching users (TSUs), as well as the beamforming vector associated with PSUs and TSUs. A two-layer algorithm based on semi-definite relaxation is proposed to tackle the complexity issue of the non-convex optimization problem. The global optimality is theoretically analyzed, and the impact of each parameter on system performance is also discussed. Numerical results indicate that TSUs are more prone to saturation compared to PSUs under identical EH requirements. The minimal required transmit power under the nonlinear EH model is much lower than that under the linear EH model. Moreover, it is observed that the number of TSUs is the primary limiting factor for the minimization of transmit power, which can be effectively mitigated by the proposed algorithm.

## 1. Introduction

Simultaneous wireless information and power transmission (SWIPT) has been envisioned as a promising solution to extend the lifespan of energy-constrained devices in the Internet of Things (IoT) networks [1,2,3]. This technology allows mobile terminals to harvest energy from electromagnetic (EM) waves transmitted through radio frequency (RF) [4]. To balance the performance of energy harvesting (EH) and information transmission, schemes like time switching (TS) and power splitting (PS) have been suggested to separate signals for information decoding (ID) and EH [5,6]. However, the transmit antenna polarization direction has great influence on the performance of EH in the SWIPT-assisted networks [7]. Integrated sensing and communication (ISAC) offers a promising solution, as sensing and communication metrics are optimized together on a single hardware unit using a unified waveform. Thus, the base station can localize users and acquire angles to each user, enabling the adjustment of the transmit antenna polarization direction [8,9].

Whereas ISAC can enable precise EH, power consumption remains a challenge for the SWIPT-assisted IoT networks. The authors in [9] investigated a sensing-assisted SWIPT network in the presence of location uncertainty, where each time frame was divided into wireless power transfer (WPT) and sensing phases, via a time-splitting strategy. In the presence of location uncertainty, the authors proposed a two-layer optimization framework to minimize the power consumption. The authors in [10] proposed a dynamic on–off control strategy to flexibly enable or disable energy transmission to minimize the power consumption. The work in [11] investigated a SWIPT-assisted ISAC network where information and energy receivers were co-located, aiming to enhance sensing performance. To achieve a balance between communication and EH, the authors jointly optimized the PS factors and beamforming vectors. In addition, the work in [12] investigated a multi-antenna system comprised of an ID receiver, an EH receiver, and a sensing target, revealing the performance trade-offs among communication, energy transfer, and sensing.

**Figure 1:**
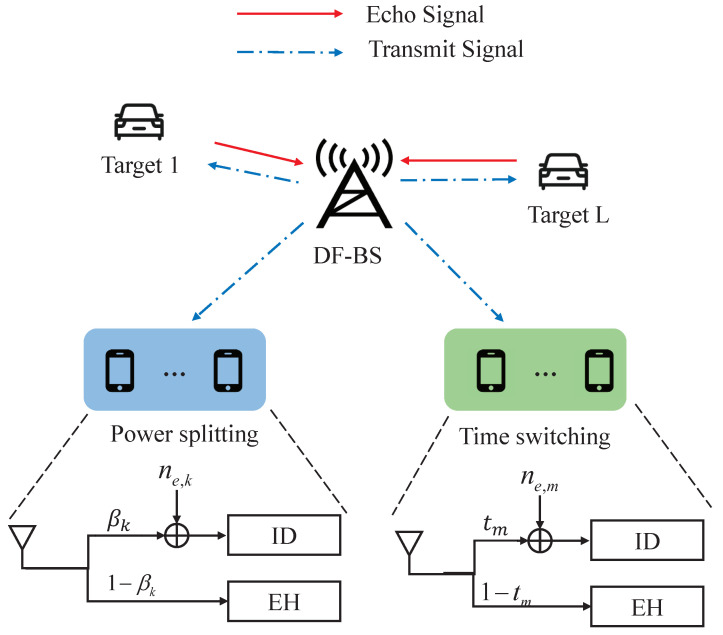
SWIPT-assisted system with ISAC.

However, an increasing number of new IoT applications like extended reality, smart cities, digital twins, and autonomous vehicles require the coexistence of PS users (PSUs) and TS users (TSUs) [13]. Concretely, the hybrid system is shown in Figure 1, where a dual-function base station (DF-BS) is utilized to transmit information and energy to multiple PSUs and TSUs and senses potential targets. The transmit power of the BS increases significantly due to the fact that the sensing signals are transferred in addition to the communication data. Moreover, the coupling of PS and TS factors makes it rather complicated for the power allocation at the BS. The above studies did not consider these problems, make it rather challenging and necessary to optimize the power consumption in such new hybrid IoT networks [14].

Motivated by the above discussion, we formulate an optimization problem for SWIPT-assisted IoT networks with ISAC to minimize the transmit power of the BS under a nonlinear EH model. The coexistence of multiple PSUs and TSUs are considered, together with the communication, EH constraints, and the sensing of the targets. To solve the non-convex optimization problem, we propose a two-layer algorithm using semi-definite relaxation (SDR) technology. The main contributions of this paper are summarized as follows:

- We consider a SWIPT-assisted system with ISAC, where the MF-BS transmits integrated sensing, communication, and energy signals to PSUs, TSUs, and targets simultaneously. We also formulate an optimization problem aimed at minimizing the required transmit power, which involves the beamforming vectors at the MF-BS, the PS factors at PSUs, the TS factors at TSUs, and the covariance matrix of sensing.
- Due to the coupling of optimization variables and the non-convexity of the nonlinear EH model, it is difficult to solve the formulated problem. To this end, we initially derive an equivalent problem by introducing auxiliary variables and SDR technology. Then, we propose a two-layer algorithm to solve the equivalent problem.
- The global optimality is theoretically analyzed, and simulation results validate the effectiveness of the proposed algorithm. In addition, simulation results show that TSUs are more likely to enter into the saturation region compared with PSUs. The minimal required transmit power under the nonlinear EH model is much lower than that under the linear EH model.

Notations: Vectors are denoted by boldface lowercase letters, while matrices are denoted by boldface uppercase letters. The symbol ∥·∥ represents the Euclidean norm operator, and trace(X) denotes the trace of a matrix X. Moreover, rank(X) represents the rank of X, and X is positive semi-definite. exp(·) denotes the exponent.

## 2. System Model

We consider a SWIPT-assisted system that comprises a DF-BS with *N* antennas forming a uniform linear array (ULA). There are *K* PSUs and *M* TSUs, respectively. All UEs are assumed to have a single antenna, and the number of sensing targets is *L*. Each UE obtains energy from signals sent by the BS. UEs with a PS receiver are denoted as ${\mathrm{PSU}}_{k}$, k=1,2,⋯,K, and UEs with a TS receiver are denoted as ${\mathrm{TSU}}_{m}$, m=1,2,⋯,M. It is assumed that the system deploys a block fading channel model, where channel coefficients remain constant within each fading block. The channels from the BS to $k^{\mathrm{th}}$ PSU and $m^{\mathrm{th}}$ TSU are defined as $h_{k}\in C^{N\times 1}$ and $g_{m}\in C^{N\times 1}$, respectively. Assume the time period of a block is normalized to one in the sequel; the transmitted ISAC signal over the whole block is

(1) $$s=\sum_{k=1}^{K}w_{k}s_{k}+\sum_{m=1}^{M}v_{m}s_{m}$$

where $w_{k}\in C^{N\times 1}$, and $v_{m}\in C^{N\times 1}$ stand for the beamforming vector associated with PSUs and TSUs, respectively. $s_{k}^{c}$ and $s_{m}^{c}$ are the information symbols for PSUs and TSUs with unit power, i.e., $E\left\{{∥s_{k}∥}^{2}\right\}=E\left\{{∥s_{m}∥}^{2}\right\}=1$ [15]. The transmit power at the BS is

(2) $$\zeta \left(w_{k},v_{m}\right)=\sum_{k=1}^{K}{∥w_{k}∥}^{2}+\sum_{m=1}^{M}{∥v_{m}∥}^{2}$$

### 2.1. Nonlinear EH Model

Due to the presence of nonlinear components such as diodes, resistors, and capacitors in the rectifier [16], we adopt the logistic-function-based nonlinear EH model, i.e.,

(3) $$E_{\mathrm{NLr}}\left(P_{\mathrm{in}}\right)=\frac{\frac{P_{\max}}{1+\exp(-a\left(P_{\mathrm{in}}-b\right))}-\frac{P_{\max}}{1+\exp(ab)}}{1-\frac{1}{1+\exp(ab)}}$$

which is given by [17]. $P_{\max}$, *a*, and *b* are in the EH circuit. $P_{\mathrm{in}}$ represents the received RF power at the energy receiver. $P_{\max}$ represents the maximum harvested energy at receiver saturation. *a* is associated with the nonlinear EH rate, and *b* reflects the EH circuit’s minimum turn-on voltage. We note that the proposed non-linear EH model is able to capture the joint effect of the non-linear phenomena caused by hardware constraints including circuit sensitivity limitations and current leakage [18,19].

### 2.2. Achievable Communication Rate and Harvested Energy

#### 2.2.1. PSUs

The received signals at ${\mathrm{PSU}}_{k}$ from the BS are divided into the ID part and the EH part via a PS factor $\beta_{k}\in \left(0,1\right)$. Therefore, the received signals at ${\mathrm{PSU}}_{k}$ can be formulated as

(4) $$\begin{array}{cc} y_{k}^{\mathrm{PSU}} & =\underset{\mathrm{Desired}\;\mathrm{signal}\;\mathrm{at}\;{\mathrm{PSU}}_{k}}{\underset{︸}{\sqrt{\beta_{k}}h_{k}^{H}w_{k}s_{k}}}+\underset{\mathrm{Interference}\;\mathrm{caused}\;\mathrm{by}\;\mathrm{the}\;\mathrm{rest}\;\mathrm{PSUs}}{\underset{︸}{\sum_{\begin{array}{c} j\neq k \end{array}}^{K}\sqrt{\beta_{k}}h_{k}^{H}w_{j}s_{j}}}+\underset{\mathrm{Interference}\;\mathrm{caused}\;\mathrm{by}\;\mathrm{TSUs}}{\underset{︸}{\sum_{\begin{array}{c} m=1 \end{array}}^{M}\sqrt{\beta_{k}}h_{k}^{H}v_{m}s_{m}}}\\ \; & +\underset{\mathrm{Noise}}{\underset{︸}{\sqrt{\beta_{k}}n_{c,k}+n_{e,k}}} \end{array}$$

where $n_{c,k}$ is the receiving antenna additive white Gaussian noise (AWGN) with zero mean and variance $\sigma_{c,k}^{2}$. $n_{e,k}∼\mathrm{CN}\left(0,\sigma_{e,k}^{2}\right)$ is the additional noise introduced by the ID at ${\mathrm{PSU}}_{k}$. Thus, the received SINR at ${\mathrm{PSU}}_{k}$ can be expressed as

(5) $$\begin{array}{c} {\mathrm{SINR}}_{k}^{\mathrm{PS}}=\frac{\beta_{k}{\left|h_{k}^{H}w_{k}\right|}^{2}}{\beta_{k}\left(\sum_{j\neq k}^{K}{\left|h_{k}^{H}w_{j}\right|}^{2}+\sum_{\begin{array}{c} m=1 \end{array}}^{M}{\left|h_{k}^{H}v_{m}\right|}^{2}+\sigma_{c,k}^{2}\right)+\sigma_{e,k}^{2}} \end{array}$$

where the signals from other PSUs, all TSUs, and targets are considered as interference. The achievable communication rate at ${\mathrm{PSU}}_{k}$ can be formulated as

(6) $$R_{k}^{\mathrm{PS}}\left(\beta_{k},w_{k},v_{m}\right)=\log\left(1+{\mathrm{SINR}}_{k}^{\mathrm{PS}}\right)$$

Meanwhile, the $\left(1-\beta_{k}\right)$ part of the received signal can be written as

(7) $$y_{k}^{\mathrm{EH}}=\sqrt{1-\beta_{k}}\left(h_{k}^{H}s+n_{c,k}\right)$$

Since the noise power of $n_{c,k}$ is small enough compared to the received signal power [20,21], the received RF power for EH at ${\mathrm{PSU}}_{k}$ is given by

(8) $$E_{k}^{\mathrm{PS}}=\left(1-\beta_{k}\right)\left(\sum_{\begin{array}{c} j=1 \end{array}}^{K}{\left|h_{k}^{H}w_{j}\right|}^{2}+\sum_{\begin{array}{c} m=1 \end{array}}^{M}{\left|h_{k}^{H}v_{m}\right|}^{2}\right)$$

By combining Equation (3), the EH at ${\mathrm{PSU}}_{k}$ within a time block can be written as

(9) $${\mathrm{EH}}_{k}^{\mathrm{PS}}=E_{\mathrm{NLr}}\left(E_{k}^{\mathrm{PS}}\right)$$

#### 2.2.2. TSUs

For ${\mathrm{TSU}}_{m}$, each time block is divided into two orthogonal time slots via a TS factor $t_{m}\in \left(0,1\right)$. The first time slot, with an interval of $t_{m}$, is dedicated to ID, while the second time slot, with an interval of $\left(1-t_{m}\right)$, is used for EH.

The received signals for ID at ${\mathrm{TSU}}_{m}$ can be formulated as

(10) $$\begin{array}{c} y_{m}^{\mathrm{TSU}}=\underset{\mathrm{Desired}\;\mathrm{signal}\;\mathrm{at}\;{\mathrm{TSU}}_{m}}{\underset{︸}{g_{m}^{H}v_{m}s_{m}}}+\underset{\mathrm{The}\;\mathrm{rest}\;\mathrm{TSUs}\;\mathrm{interference}}{\underset{︸}{\sum_{\begin{array}{c} i\neq m \end{array}}^{M}g_{m}^{H}v_{i}s_{i}}}+\underset{\mathrm{PSUs}\;\mathrm{interference}}{\underset{︸}{\sum_{\begin{array}{c} k=1 \end{array}}^{K}g_{m}^{H}w_{k}s_{k}}}+\underset{\mathrm{Noise}}{\underset{︸}{n_{c,m}+n_{e,m}}} \end{array}$$

where $n_{c,m}∼\mathrm{CN}\left(0,\sigma_{c,m}^{2}\right)$. The term $n_{e,m}∼\mathrm{CN}\left(0,\sigma_{e,m}^{2}\right)$ represents RF-to-baseband conversion noise. Therefore, the received SINR at ${\mathrm{TSU}}_{m}$ can be written as

(11) $$\begin{array}{cc} \; & {\mathrm{SINR}}_{m}^{\mathrm{TS}}=\frac{{\left|g_{m}^{H}v_{m}\right|}^{2}}{\sum_{i\neq m}^{M}{\left|g_{m}^{H}v_{i}\right|}^{2}+\sum_{\begin{array}{c} k=1 \end{array}}^{K}{\left|g_{m}^{H}w_{k}\right|}^{2}+\sigma_{c,m}^{2}+\sigma_{e,m}^{2}} \end{array}$$

The achievable communication rate at ${\mathrm{TSU}}_{m}$ can be formulated as

(12) $$R_{m}^{\mathrm{TS}}\left(t_{m},w_{k},v_{m}\right)=t_{m}\log\left(1+{\mathrm{SINR}}_{m}^{\mathrm{TS}}\right)$$

In the second time slot, the received RF energy for EH at ${\mathrm{TSU}}_{m}$ can be given by

(13) $$E_{m}^{\mathrm{TS}}=\left(\sum_{\begin{array}{c} k=1 \end{array}}^{K}{\left|g_{m}^{H}w_{k}\right|}^{2}+\sum_{\begin{array}{c} i=1 \end{array}}^{M}{\left|g_{m}^{H}v_{i}\right|}^{2}\right)$$

By combining with Equation (3), the EH at ${\mathrm{TSU}}_{m}$ within the second time slot can be written as

(14) $${\mathrm{EH}}_{m}^{\mathrm{TS}}=\left(1-t_{m}\right)E_{\mathrm{NLr}}\left(E_{m}^{\mathrm{TS}}\right)$$

### 2.3. Sensing Model

We consider a point target model and assume that the radar channel consists of line-of-sight (LoS) paths, with both transmit and receive ULAs at the BS having half-wavelength antenna spacing. The angle of departure (AOD) and angle of arrival (AOA) of the target are represented by $0\leq \theta_{t}\leq 2\pi$ and $0\leq \theta_{r}\leq 2\pi$, respectively. $a\left(\theta_{t}\right)\in C^{N\times 1}$ and $a\left(\theta_{r}\right)\in C^{N\times 1}$ denote the transmit and receive array steering vectors, respectively. We assume equal angles of arrival and departure for the target, i.e., $\theta_{r}=\theta_{t}=\theta$, due to our consideration of a monostatic radar setting [22,23]. Thus, we can express $a\left(\theta_{t}\right)=a\left(\theta_{r}\right)$ using

(15) $$a\left(\theta \right)=\left[1,e^{j\pi \sin\theta },\cdots ,e^{j\pi \left(N-1\right)\sin\theta }\right]$$

Following [22], the target response matrix is expressed as

(16) $$T_{s}=\alpha a\left(\theta \right)a^{H}\left(\theta \right)\overset{\Delta }{\;=}\alpha A\left(\theta \right)$$

where α∈C is the complex amplitude of the target mainly determined by the path loss and the radar cross section [24].

Thus, the reflected echo signal by the target is denoted by $Y_{R}\in C^{N\times T}$, which can be expressed as

(17) $$Y_{R}=T_{s}s+N_{R}$$

where $N_{R}$ is the AWGN matrix with the variance of each entry being $\sigma_{R}$. With the prior information, the power of the probing signal in target directions can be formulated as

(18) $$P\left(\theta_{l}\right)={a\left(\theta_{l}\right)}^{H}R_{s}a\left(\theta_{l}\right),\;l=1,2,\cdots ,L$$

where $R_{s}=\sum_{\begin{array}{c} k=1 \end{array}}^{K}{w_{k}w_{k}}^{H}+\sum_{\begin{array}{c} m=1 \end{array}}^{M}v_{m}v_{m}^{H}$ represents the sample covariance matrix of the transmitted signal.

To ensure similar levels of sensing power in different target directions, i.e., $\theta_{p}$ and $\theta_{q}$, the difference of $P\left(\theta_{p}\right)$ and $P\left(\theta_{q}\right)$ is assumed to be low [25] and is written as

(19) $$\left|P\left(\theta_{p}\right)-P\left(\theta_{q}\right)\right|,\;\forall p\neq q\in \left\{1,2,\cdots ,L\right\}$$

By setting similar levels of sensing power in different directions, we can ensure that targets in all directions are fairly tracked. In particular, the sensing power of the target is equal when Equation (19) is equal to zero.

## 3. Problem Formulation

We aim to minimize the BS transmit power while meeting the communication rate and EH requirements of all users, as well as radar-specific requirements. The optimization problem can be formulated as

(20) $$\begin{array}{cc} \left(P1\right):\; & \min_{w_{k},v_{m},\beta_{k},t_{m}}\;\zeta \left(w_{k},v_{m}\right) \end{array}$$

(21) $$\begin{array}{cc} s.t.\; & R_{k}^{\mathrm{PS}}\left(\beta_{k},w_{k},v_{m}\right)\geq \gamma_{k}^{\mathrm{PS}},\;\forall k \end{array}$$

(22) $$\begin{array}{cc} \; & {\mathrm{EH}}_{k}^{\mathrm{PS}}\geq \eta_{k}^{\mathrm{PS}},\;\forall k \end{array}$$

(23) $$\begin{array}{cc} \; & R_{m}^{\mathrm{TS}}\left(t_{m},w_{k},v_{m}\right)\geq \gamma_{m}^{\mathrm{TS}},\;\forall m \end{array}$$

(24) $$\begin{array}{cc} \; & {\mathrm{EH}}_{m}^{\mathrm{TS}}\geq \eta_{m}^{\mathrm{TS}},\;\forall m \end{array}$$

(25) $$\begin{array}{cc} \; & \left|P\left(\theta_{p}\right)-P\left(\theta_{q}\right)\right|\leq P_{\mathrm{diff}},\;\forall p\neq q\in \left\{1,2,\cdots ,L\right\} \end{array}$$

(26) $$\begin{array}{cc} \; & 0<\beta_{k},t_{m}<1,\;\forall k,m \end{array}$$

where $\gamma_{k}^{\mathrm{PS}}$ and $\gamma_{m}^{\mathrm{TS}}$ represent the minimum communication rate requirements for ${\mathrm{PSU}}_{k}$ and ${\mathrm{TSU}}_{m}$, respectively. Similarly, $\eta_{k}^{\mathrm{PS}}$ and $\eta_{m}^{\mathrm{TS}}$ denote the EH thresholds for ${\mathrm{PSU}}_{k}$ and ${\mathrm{TSU}}_{m}$, respectively. Here, (21) and (23) ensure the minimum rate for each UE, while (22) and (24) ensure the minimum EH requirements. In addition, (25) guarantees similar levels of sensing power in different target directions, where $P_{\mathrm{diff}}$ is the requirement for the minimum difference in sensing power among these directions.

However, the problem (P1) is non-convex due to the presence of coupled variables in the constraints and quadratic terms involving $w_{k}$ and $v_{m}$ in the objective function and constraints, which cannot be directly solved. To reformulate (P1) into a tractable form, we first introduce a set of auxiliary matrix variables, including $W_{k}=w_{k}{w_{k}}^{H}$ with $\mathrm{rank}\left(W_{k}\right)=1$ and $V_{m}=v_{m}{v_{m}}^{H}$ with $\mathrm{rank}\left(V_{m}\right)=1$. Thus, we have ${\left|g_{m}^{H}v_{m}\right|}^{2}=g_{m}^{H}V_{m}g_{m}$, ${\left|g_{m}^{H}w_{k}\right|}^{2}=g_{m}^{H}W_{k}g_{m}$, ${\left|h_{k}^{H}w_{k}\right|}^{2}=h_{k}^{H}W_{k}h_{k}$, and ${\left|h_{k}^{H}v_{m}\right|}^{2}=h_{k}^{H}V_{m}h_{k}$

The constraints (21) and (23) can be reformulated as

(27) $$\begin{array}{c} {\left|h_{k}^{H}w_{k}\right|}^{2}\geq \left(2^{\gamma_{k}^{\mathrm{PS}}}-1\right)\times \left(\left(\sum_{j\neq k}^{K}{\left|h_{k}^{H}w_{j}\right|}^{2}+\sum_{\begin{array}{c} m=1 \end{array}}^{M}{\left|h_{k}^{H}v_{m}\right|}^{2}+\sigma_{c,k}^{2}\right)+\frac{\sigma_{e,k}^{2}}{\sigma_{k}}\right) \end{array}$$

(28) $$\begin{array}{c} {\left|g_{m}^{H}v_{m}\right|}^{2}\geq \left(2^{\gamma_{m}^{\mathrm{TS}}}-1\right)\times \left(\sum_{i\neq m}^{M}{\left|g_{m}^{H}v_{i}\right|}^{2}+\sum_{\begin{array}{c} k=1 \end{array}}^{K}{\left|g_{m}^{H}w_{k}\right|}^{2}+\sigma_{c,m}^{2}+\sigma_{e,m}^{2}\right) \end{array}$$

With the auxiliary matrix variables, (21) and (23) can be further rewritten as

(29) $$\begin{array}{c} h_{k}^{H}W_{k}h_{k}\geq \left(2^{\gamma_{k}^{\mathrm{PS}}}-1\right)\times \left(\left(\sum_{j\neq k}^{K}h_{k}^{H}W_{j}h_{k}+\sum_{\begin{array}{c} m=1 \end{array}}^{M}h_{k}^{H}V_{m}h_{k}+\sigma_{c,k}^{2}\right)+\frac{\sigma_{e,k}^{2}}{\beta_{k}}\right) \end{array}$$

(30) $$\begin{array}{c} g_{m}^{H}V_{m}g_{m}\geq \left(2^{\gamma_{m}^{\mathrm{TS}}/t_{m}}-1\right)\times \left(\sum_{i\neq m}^{M}g_{m}^{H}V_{i}g_{m}+\sum_{\begin{array}{c} k=1 \end{array}}^{K}g_{m}^{H}W_{k}g_{m}+\sigma_{c,m}^{2}+\sigma_{e,m}^{2}\right) \end{array}$$

Moreover, the inverse function of (3) can be written as

(31) $$P_{\mathrm{in}}\left(E_{\mathrm{NLr}}\right)=b-\frac{1}{a}\ln\left(\frac{\left(P_{\max}-E_{\mathrm{NLr}}\right)\exp\left(ab\right)}{P_{\max}+E_{\mathrm{NLr}}\exp\left(ab\right)}\right)$$

**Remark** **1.** *According to* (31), $P_{\max}>E_{\mathrm{NLr}}$. *Consequently, the following constraints apply:*

(32) $$\begin{array}{c} \eta_{m}^{\mathrm{TS}}<\left(1-t_{m}\right)P_{\max} \end{array}$$

(33) $$\begin{array}{c} \eta_{m}^{\mathrm{PS}}<P_{\max} \end{array}$$

*Thus, $t_{m}$ is within $\left(0,1-\frac{\eta_{m}^{\mathrm{TS}}}{P_{\max}}\right)$.*

The constraints (22) and (24) can be reformulated as

(34) $$\sum_{\begin{array}{c} j=1 \end{array}}^{K}h_{k}^{H}W_{j}h_{k}+\sum_{\begin{array}{c} m=1 \end{array}}^{M}h_{k}^{H}V_{m}h_{k}\geq \frac{P_{\mathrm{in}}\left(\eta_{k}^{\mathrm{PS}}\right)}{1-\beta_{k}}$$

(35) $$\sum_{\begin{array}{c} k=1 \end{array}}^{K}g_{m}^{H}W_{k}g_{m}+\sum_{\begin{array}{c} i=1 \end{array}}^{M}g_{m}^{H}V_{i}g_{m}\geq P_{\mathrm{in}}\left(\frac{\eta_{m}^{\mathrm{TS}}}{1-t_{m}}\right)$$

By unifying (29), (30), (34), and (35), the problem (P1) can be equivalently rewritten as

(36) $$\begin{array}{cc} \left(P2\right):\; & \min_{W_{k},V_{m},\beta_{k},t_{m}}\;\zeta \left(W_{k},V_{m}\right)=\sum_{k=1}^{K}\mathrm{Tr}\left(W_{k}\right)+\sum_{m=1}^{M}\mathrm{Tr}\left(V_{m}\right) \end{array}$$

(37) $$\begin{array}{cc} s.t.\; & \left(29),(30),(34),(35),(25)(26\right) \end{array}$$

(38) $$\begin{array}{cc} \; & W_{k}⪰0,V_{m}⪰0 \end{array}$$

(39) $$\begin{array}{cc} \; & \mathrm{rank}\left(W_{k}\right)=1,\mathrm{rank}\left(V_{m}\right)=1 \end{array}$$

The rank-one constraints in (39) can be ignored by using the SDR method [26,27]. Therefore, problem (P2) can be rewritten as

(40) $$\begin{array}{cc} \left(P3\right):\; & \min_{W_{k},V_{m},\beta_{k},t_{m}}\;\zeta \left(W_{k},V_{m}\right) \end{array}$$

(41) $$\begin{array}{cc} s.t.\; & (37),(38),\;\forall m,k \end{array}$$

Since $V_{m}$ and $t_{m}$ are coupled, problem (P3) remains non-convex. However, when $t_{m}$ is fixed, problem (P3) becomes solvable. As such, we propose an algorithm based on the interior-point method [26] to address problem (P3), as outlined in Algorithm 1. In the inner layer, SDR is applied. The optimal set $\left\{W_{k}^{*},V_{m}^{*},\beta_{k}^{*}\right\}$ is obtained by solving the relaxed convex problem (P3) for a given set of $\left\{t_{m}\right\}$.

Problems (P2) and (P3) are equivalent whenever $\mathrm{rank}\left(W_{k}\right)=1$, $\mathrm{rank}\left(V_{m}\right)=1$. The optimal sets $\left\{w_{k}^{*}\right\}$ and $\left\{v_{m}^{*}\right\}$ of the original problem (P1) can be obtained by the eigenvalue decomposition (EVD) of the optimal sets $\left\{W_{k}^{*}\right\}$ and $\left\{V_{m}^{*}\right\}$, respectively. In the outer layer, a 1-D search is utilized to determine the optimal set $\left\{t_{m}^{*}\right\}$. By exhaustive searching, the optimal solution can be attained. Once $\left\{W_{k}^{*},V_{m}^{*},\beta_{k}^{*},t_{m}^{*}\right\}$ is obtained, the optimal set $\left\{w_{k}^{*},v_{m}^{*},\beta_{k}^{*},t_{m}^{*}\right\}$ of problem (P1) can be derived. The computational complexity of Algorithm 1 is high, due to the 1-D search. Thus, we propose a BiS algorithm based on the bisection method, i.e., Algorithm 2.

Next, we theoretically analyze the impact of the communication rate and EH requirements on the system performance for TSUs and PSUs, respectively.

For the communication rate requirements of TSUs and PSUs, considering that $0<t_{m}<\left(1-\eta_{m}^{\mathrm{TS}}/P_{\max}\right)<1$, we can deduce that $\left(2^{\gamma_{k}^{\mathrm{PS}}}-1\right)<\left(2^{\gamma_{m}^{\mathrm{TS}}/t_{m}}-1\right)$ when $\gamma_{k}^{\mathrm{PS}}=\gamma_{m}^{\mathrm{TS}}$. Therefore, for the constraints (29) and (30), we can conclude that the communication rate requirement threshold, $\gamma_{k}^{\mathrm{PS}}$, has an impact on the transmit power compared to $\gamma_{m}^{\mathrm{TS}}$. In terms of the EH requirements at TSUs and PSUs, if $\eta_{k}^{\mathrm{PS}}=\eta_{m}^{\mathrm{TS}}$ and (23) and (24) are both satisfied with equality, then we can conclude that ${\mathrm{EH}}_{k}^{\mathrm{PS}}={\mathrm{EH}}_{m}^{\mathrm{TS}}$, i.e.,

(42) $$E_{\mathrm{NLr}}\left(E_{k}^{\mathrm{PS}}\right)=\left(1-t_{m}\right)E_{\mathrm{NLr}}\left(E_{m}^{\mathrm{TS}}\right)$$

**Algorithm 1** Two-layer algorithm1:**Input:** $\gamma_{k}^{\mathrm{PS}}$, $\gamma_{m}^{\mathrm{TS}}$, $\eta_{k}^{\mathrm{PS}}$, $\eta_{m}^{\mathrm{TS}}$, the search step size δ.2:**for** each $t_{m}\in \left(0,1\right)$ with δ **do**3:   Calculate $\left\{W_{k}^{*},V_{m}^{*},\beta_{k}^{*}\right\}$ by solving problem (P3) using CVX.4:**end for**5:Find the optimal set $\left\{t_{m}^{*}\right\}$6:According to $\left\{W_{k}^{*},V_{m}^{*},\beta_{k}^{*}\right\}$, calculate $\left\{w_{k}^{*},v_{m}^{*},\beta_{k}^{*}\right\}$ by EVD.7:**Output:** The optimal set $\left\{w_{k}^{*},v_{m}^{*},\beta_{k}^{*},t_{m}^{*}\right\}$ and the minimal transmit power $\zeta \left(w_{k}^{*},v_{m}^{*}\right)$.

**Algorithm 2** BiS algorithm
1:**Input:** $\gamma_{k}^{\mathrm{PS}}$, $\gamma_{m}^{\mathrm{TS}}$, $\eta_{k}^{\mathrm{PS}}$, $\eta_{m}^{\mathrm{TS}}$, the solution accuracy $\delta_{\mathrm{bs}}$.2:Set the initial lower and upper bounds for problem (P3), i.e., $t_{m}^{\mathrm{low}}$ and $t_{m}^{\mathrm{upp}}$.3:**while** $(t_{m}^{\mathrm{low}}-t_{m}^{\mathrm{upp}})>\delta$ **do**4:   $t_{m}^{\mathrm{mid}}\leftarrow \frac{\left(t_{m}^{\mathrm{low}}+t_{m}^{\mathrm{upp}}\right)}{2}$5:   Solve problem (P3) for fixed $t_{m}=t_{m}^{\mathrm{mid}}$.6:   **if** problem (P3) is feasible **then**7:     $t_{m}^{\mathrm{low}}\leftarrow t_{m}^{\mathrm{mid}}$8:     $\left\{W_{k}^{*},V_{m}^{*},\beta_{k}^{*}\right\}\leftarrow \left\{W_{k},V_{m},\beta_{k}\right\}$, which is the solution of problem (P3).9:   **else**10:     $t_{m}^{\mathrm{upp}}\leftarrow t_{m}^{\mathrm{mid}}$11:   **end if**12:
**end while**
13:According to $\left\{W_{k}^{*},V_{m}^{*},\beta_{k}^{*}\right\}$, calculate $\left\{w_{k}^{*},v_{m}^{*},\beta_{k}^{*}\right\}$ by EVD.14:**Output:** The optimal set $\left\{w_{k}^{*},v_{m}^{*},\beta_{k}^{*},t_{m}^{*}\right\}$ and the minimal transmit power $\zeta (w_{k}^{*},v_{m}^{*})$.


Given that $0<t_{m}<\left(1-\eta_{m}^{\mathrm{TS}}/P_{\max}\right)<1$ and the maximum energy collected by each TSU and PSU cannot exceed the saturation value of the EH circuit ($P_{\max}$), we can conclude that

(43) $$E_{\mathrm{NLr}}\left(E_{k}^{\mathrm{PS}}\right)<E_{\mathrm{NLr}}\left(E_{m}^{\mathrm{TS}}\right)\leq P_{\max}$$

Therefore, as the input RF power increases, TSUs reach the $P_{\max}$ threshold for EH before PSUs. Upon reaching this threshold, the EH circuit enters a saturation phase, as outlined in [17]. Therefore, we know TSUs are more likely to enter the saturation region of the practical EH circuit compared to PSUs with equivalent EH requirements.

Next, we analyze the complexity of Algorithm 1. Since the constraints of problem (P3) are all linear matrix inequalities when given $t_{m}$, we take the standard interior-point method to analyze the computational complexity [26]. In the outer layer, a 1-D search is utilized, with a complexity of $O\left({\left(1/\delta \right)}^{M}\right)$. In the inner layer, there are $\left(2N^{2}K\right)$ variables and $\left(3K+3M+L\right)$ linear and convex constraints in the problem. Therefore, the worst-case computational complexity of Algorithm 1 is $O\left({\left(1/\delta \right)}^{M}{\left(2N^{2}K\right)}^{3}\left(3K+3M+L\right)\right)$. For Algorithm 2, the computational complexity is $O\left(\log\left(\frac{t_{m}^{\mathrm{upp}}-t_{m}^{\mathrm{low}}}{\delta_{\mathrm{bs}}}\right)\right)$.

## 4. Numerical Results

### 4.1. Parameter Setup

We assumed that the BS had N=4 antennas serving both K=2 PSUs and M=1 TSUs, with L=2 sensing targets positioned at $40^{\circ }$ and $-40^{\circ }$, respectively. In all simulations, we assumed that all devices had the same noise power, with $\sigma_{c,m}^{2}=\sigma_{c,k}^{2}=-120$ dBm and $\sigma_{e,m}^{2}=\sigma_{e,k}^{2}=-80$ dBm. The channels between BS and users were assumed to experience Rayleigh fading with a path loss of $32.6+36.7\log_{10}\left(d\right)$ [28], where *d* represents the distance between BS and users. We set $P_{\mathrm{diff}}=10$ dBm and $\gamma_{k}^{\mathrm{PS}}=\gamma_{m}^{\mathrm{TS}}=2$ bits/s/Hz. The minimum EH requirements of PSUs and TSUs were set to $\eta_{k}^{\mathrm{PS}}=\eta_{m}^{\mathrm{TS}}=5$ J. Parameters at the EH parameters were set to a=150 and b=0.014, with the maximum harvested energy limited to $P_{\max}=30$ dB. These parameters remained unchanged unless explicitly specified otherwise.

### 4.2. Simulation Results

**Figure 2:**
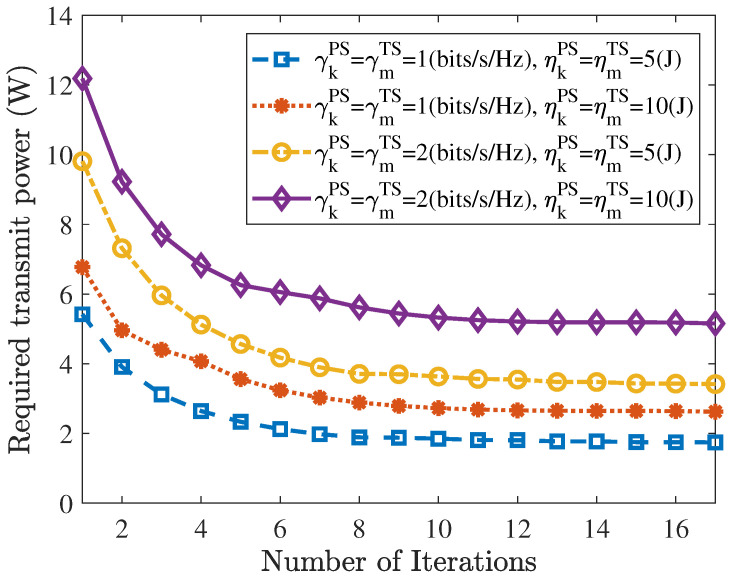
Convergence of the proposed algorithm with various information rate and harvested energy of TSUs and PSUs.

Figure 2 shows that the proposed two-layer algorithm converged within a few iterations. Moreover, Algorithm 1 maintained convergence for various combinations of $\gamma_{k}^{\mathrm{PS}}$, $\gamma_{m}^{\mathrm{TS}}$, $\eta_{m}^{\mathrm{TS}}$, and $\eta_{m}^{\mathrm{TS}}$. To discuss the effects of $\gamma_{k}^{\mathrm{PS}}$ and $\gamma_{m}^{\mathrm{TS}}$ on the minimal required transmit power, we set various combinations of $\gamma_{k}^{\mathrm{PS}}$ and $\gamma_{m}^{\mathrm{TS}}$. According to [29], when $\gamma_{m}^{\mathrm{TS}}>2$ bits/s/Hz, it had a greater impact on the required transmit power, leading to constraint (25) being infeasible. Thus, we set $\gamma_{k}^{\mathrm{PS}}$ and $\gamma_{m}^{\mathrm{TS}}$ within the range [0.2, 3] and [0.2, 2] bits/s/Hz, respectively.

**Figure 3:**
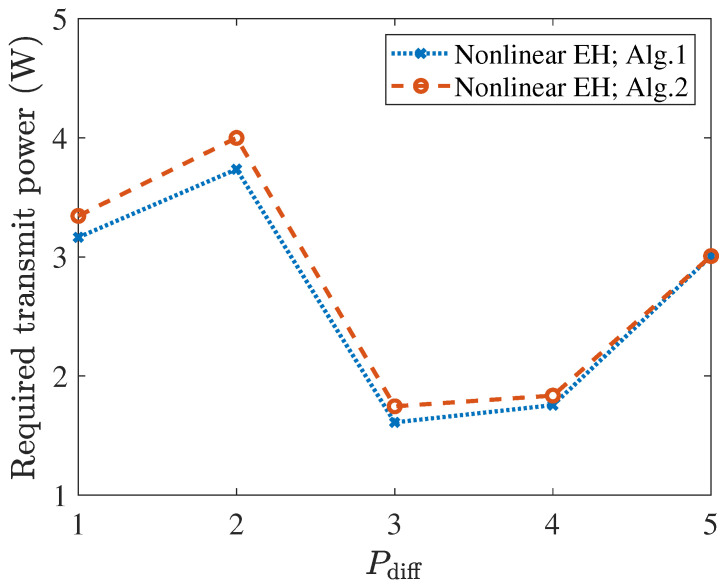
Required transmit power vs. $P_{\mathrm{diff}}$, under Algorithms 1 and 2.

**Figure 4:**
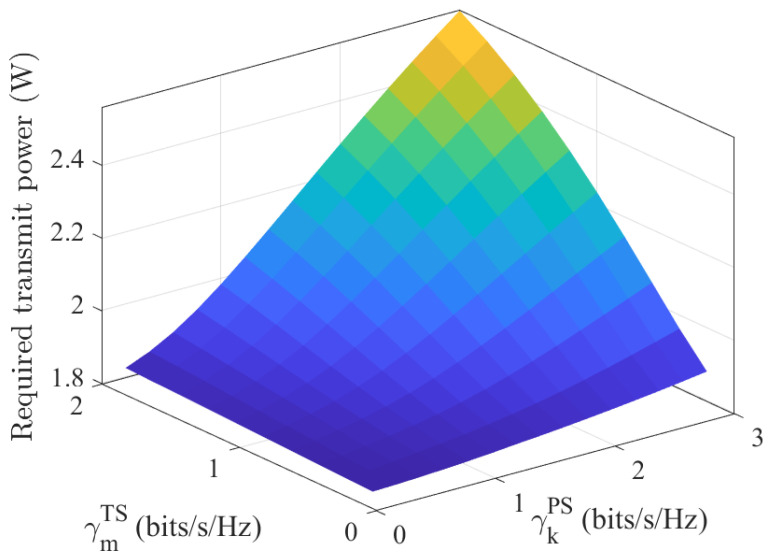
Required transmit power vs. communication rate requirements of TSUs and PSUs.

Figure 3 illustrates the required transmit power versus $P_{\mathrm{diff}}$ for Algorithms 1 and 2. Notably, Algorithm 1 consistently outperformed Algorithm 2 across the evaluated $P_{\mathrm{diff}}$ range, achieving a lower transmit power requirement at the same $P_{\mathrm{diff}}$ This performance gap widened significantly at a lower $P_{\mathrm{diff}}$. When $P_{\mathrm{diff}}=1$, the value of Algorithm 1 was 5.38% lower than the value of Algorithm 2. Meanwhile, when $P_{\mathrm{diff}}=5$, the value of Algorithm 1 was almost identical to the value of Algorithm 2, with a difference close to 0%. These results validate the superiority of Algorithm 2 in high $P_{\mathrm{diff}}$ scenarios. Figure 4 illustrates that higher communication rate requirements for both PSUs and TSUs led to an increase in the minimum transmit power. Specifically, when $\gamma_{k}^{\mathrm{PS}}=1$ bits/s/Hz and $\gamma_{m}^{\mathrm{TS}}$ changed from 1 to 2 bits/s/Hz, the corresponding change in required transmit power was smaller than when $\gamma_{m}^{\mathrm{TS}}=1$ bits/s/Hz and $\gamma_{k}^{\mathrm{PS}}$ changed from 1 to 2 bits/s/Hz. This aligned with the earlier discussion regarding the thresholds $\gamma_{k}^{\mathrm{PS}}$ for PSUs and $\gamma_{m}^{\mathrm{TS}}$ for the communication rate thresholds for TSUs. Numerical simulations validated that the former had a greater impact on the transmit power.

**Figure 5:**
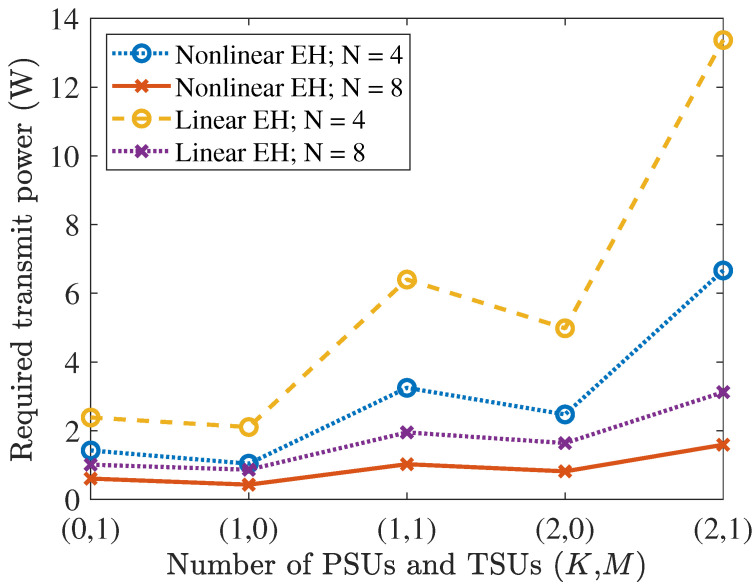
Required transmit power vs. different numbers of TSUs and PSUs with N=4 and N=8 antennas, respectively.

Figure 5 illustrates the minimal required transmit power vs. different combinations of PSUs and TSUs. Concretely, the linear EH model was $E_{\mathrm{Lr}}\left(P_{\mathrm{in}}\right)=\eta_{\mathrm{in}}P_{\mathrm{in}}$, where $\eta_{\mathrm{in}}=0.8$ is the energy conversion efficiency [29]. Notably, the gap of the required transmit power between the linear and nonlinear EH models increased with the increment of *K* and *M*, when the total number of PSUs and TSUs was same. It can be seen that with the same number of antennas, the minimum transmit power of the system was lower when there was only one user, i.e., K=1 and M=0, or K=0 and M=1. Similarly, for two users, the minimum transmit power was lower when K=1 and M=1 compared to the case when K=2 and M=0. This suggests that the minimum transmit power tends to be higher when the number of TSUs is larger. Specifically, we observed the required transmit power increased when the TSUs increased with the same number of users. This was due to all PSUs and TSUs operating at the same time in this system. This is because we set the same time for transmit information and energy harvesting for all users. As a result, TSUs were more likely to enter the saturation region of the practical EH circuit compared to PSUs, thus requiring more power within the same period, as discussed previously.

**Figure 6:**
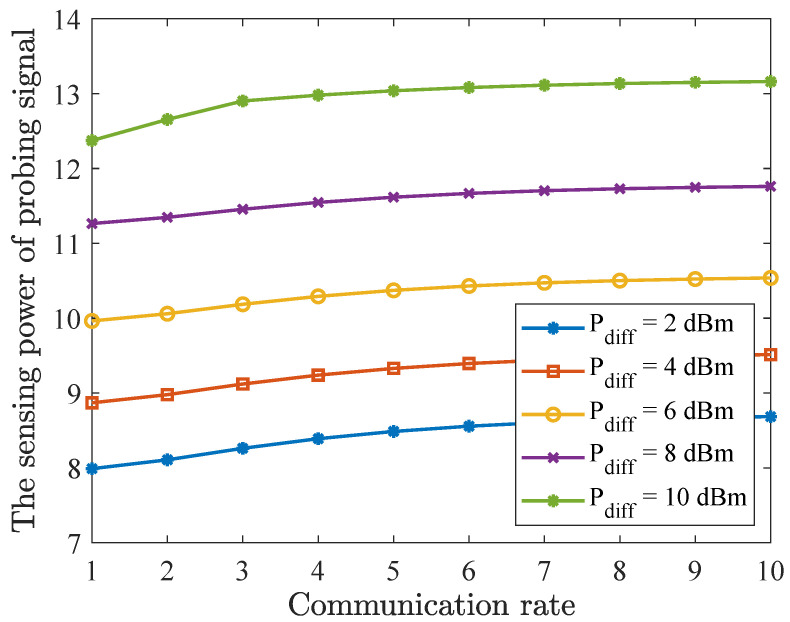
The sensing power of the probing signal versus the communication rate, with $P_{\mathrm{diff}}$ set at 2 dBm, 4 dBm, 6 dBm, 8 dBm, and 10 dBm.

In order to further explore the relationship between the user’s communication rate and the sensing power of the probing signal, Figure 6 shows the directional gain at different communication rates with varying $P_{\mathrm{diff}}$. The communication rate is set to 1–10 bit/s/Hz. Specifically, $P_{\mathrm{diff}}$ was set to 2 dBm, 4 dBm, 6 dBm, 8 dBm, and 10 dBm, respectively. The analysis of the relationship between the communication rate, $P_{\mathrm{diff}}$, and the sensing power of the probing signal revealed several key trends. As the communication rate increased from 1 bit/s/Hz to 10 bit/s/Hz, the sensing power consistently rose, indicating that higher communication rates enhance the signal quality. Furthermore, varying $P_{\mathrm{diff}}$ levels significantly impacted the sensing power; higher $P_{\mathrm{diff}}$ values led to greater sensing power at the same communication rate. This suggests that both the communication rate and $P_{\mathrm{diff}}$ play crucial roles in optimizing the sensing power of the probing signal, particularly in applications demanding high signal quality.

## 5. Conclusions

The paper investigated SWIPT-assisted IoT networks with ISAC with a focus on minimizing the transmit power of the DF-BS. An optimization problem was formulated to minimize the transmit power at the DF-BS, which considered power-splitting and time-switching factors, as well as the beamforming vector associated with PSUs and TSUs. The non-convex optimization model was solved by introducing a two-layer algorithm utilizing SDR and one-dimensional search. The global optimality was theoretically analyzed, and the impact of each parameter on system performance was also discussed. Numerical results indicated that TSUs were more prone to saturation compared to PSUs under identical EH requirements. Moreover, the minimal required transmit power under the nonlinear EH model was much lower than that under the linear EH model. Moreover, it was observed that the number of TSUs served as the primary limiting factor for minimum transmission power, which could be effectively mitigated by the proposed algorithm. Future extensions of this work will consider the scalability of the proposed algorithm in large-scale IoT systems, involving a higher number of users. Moreover, robustness against channel uncertainty will be investigated to enhance the practicality of the approach in real-world deployments.

## Data Availability

The original contributions presented in this study are included in the article. Further inquiries can be directed to the corresponding author(s).

## References

[1] Ponnimbaduge Perera T.D., Jayakody D.N.K., Sharma S.K., Chatzinotas S., Li J. (2018). Simultaneous Wireless Information and Power Transfer (SWIPT): Recent Advances and Future Challenges. IEEE Commun. Surv. Tutor..

[2] Rong B. (2021). 6G: The Next Horizon: From Connected People and Things to Connected Intelligence. IEEE Wireless Commun..

[3] Lu W., Si P., Huang G., Han H., Qian L., Zhao N., Gong Y. (2021). SWIPT Cooperative Spectrum Sharing for 6G-Enabled Cognitive IoT Network. IEEE Internet Things J..

[4] Chen X., Zhou C., Wang Z., Zhao T., Sun Q., Xu C., Zhang J. (2024). Energy Efficiency of Wireless-Powered Cell-Free mMIMO With Hardware Impairments. IEEE Trans. Commun..

[5] Zhou X., Zhang R., Ho C.K. (2013). Wireless Information and Power Transfer: Architecture Design and Rate-Energy Tradeoff. IEEE Trans. Commun..

[6] Shi Q., Liu L., Xu W., Zhang R. (2014). Joint Transmit Beamforming and Receive Power Splitting for MISO SWIPT Systems. IEEE Trans. Wireless Commun..

[7] Li W., Yu Q., Qiu J., Qi J. (2024). Intelligent wireless power transfer via a 2-bit compact reconfigurable transmissive-metasurface-based router. Nat. Commun.

[8] Wu N., Jiang R., Wang X., Yang L., Zhang K., Yi W., Nallanathan A. (2024). AI-enhanced integrated sensing and communications: Advancements, challenges, and prospects. IEEE Commun. Mag..

[9] Xu Y., Xu D., Song S. (2024). Sensing-assisted Robust SWIPT for Mobile Energy Harvesting Receivers in Networked ISAC Systems. IEEE Trans. Wireless Commun..

[10] Hao Z., Fang Y., Yu X., Xu J., Qiu L., Xu L., Cui S. (2025). Energy-Efficient Hybrid Beamforming With Dynamic On-Off Control for Integrated Sensing, Communications, and Powering. IEEE Trans. Commun..

[11] Zhou Z., Li X., Zhu G., Xu J., Huang K., Cui S. (2024). Integrating Sensing, Communication, and Power Transfer: Multiuser Beamforming Design. IEEE J. Sel. Areas Commun..

[12] Chen Y., Hua H., Xu J., Ng D.W.K. (2024). ISAC Meets SWIPT: Multi-Functional Wireless Systems Integrating Sensing, Communication, and Powering. IEEE Trans. Wireless Commun..

[13] Cui Y., Liu F., Jing X., Mu J. (2021). Integrating sensing and communications for ubiquitous IoT: Applications, trends, and challenges. IEEE Netw..

[14] Mittelbach M., Schaefer R.F., Bloch M., Yener A., Günlü O. (2025). Sensing-Assisted Secure Communications over Correlated Rayleigh Fading Channels. Entropy.

[15] Li X., Yi X., Zhou Z., Han K., Han Z., Gong Y. Multi-User Beamforming Design for Integrating Sensing, Communications, and Power Transfer. Proceedings of the 2023 IEEE Wireless Communications and Networking Conference (WCNC).

[16] Shi E., Zhang J., Chen S., Zheng J., Zhang Y., Kwan Ng D.W., Ai B. (2022). Wireless Energy Transfer in RIS-Aided Cell-Free Massive MIMO Systems: Opportunities and Challenges. IEEE Commun. Mag..

[17] Boshkovska E., Ng D.W.K., Zlatanov N., Schober R. (2015). Practical Non-Linear Energy Harvesting Model and Resource Allocation for SWIPT Systems. IEEE Commun. Lett..

[18] Le T., Mayaram K., Fiez T. (2008). Efficient Far-Field Radio Frequency Energy Harvesting for Passively Powered Sensor Networks. IEEE J. Solid-State Circuits..

[19] Guo J., Zhu X. An improved analytical model for RF-DC conversion efficiency in microwave rectifiers. Proceedings of the 2012 IEEE/MTT-S International Microwave Symposium Digest.

[20] Lu Y., Xiong K., Fan P., Zhong Z., Ai B., Letaief K.B. (2022). Worst-Case Energy Efficiency in Secure SWIPT Networks with Rate-Splitting ID and Power-Splitting EH Receivers. IEEE Trans. Wireless Commun..

[21] Shi Q., Peng C., Xu W., Hong M., Cai Y. (2016). Energy Efficiency Optimization for MISO SWIPT Systems with Zero-Forcing Beamforming. IEEE Trans. Signal Process..

[22] Bekkerman I., Tabrikian J. (2006). Target Detection and Localization Using MIMO Radars and Sonars. IEEE Trans. Signal Process..

[23] Ahmadipour M., Wigger M., Shamai S. (2025). Exploring ISAC: Information-Theoretic Insights. Entropy.

[24] Pritzker J., Ward J., Eldar Y.C. Transmit Precoding for Dual-Function Radar-Communication Systems. Proceedings of the 2021 55th Asilomar Conference on Signals, Systems, and Computers.

[25] Stoica P., Li J., Xie Y. (2007). On Probing Signal Design for MIMO Radar. IEEE Trans. Signal Process..

[26] Boyd S., Vandenberghe L. (2004). Convex Optimization.

[27] Chen Y., Hua H., Xu J. Transmit Optimization for Multi-functional MIMO Systems Integrating Sensing, Communication, and Powering. Proceedings of the ICC 2023—IEEE International Conference on Communications.

[28] Wang Z., Mu X., Liu Y., Xu X., Zhang P. (2022). NOMA-aided joint communication, sensing, and multi-tier computing systems. IEEE J. Sel. Areas Commun..

[29] Jiang R., Xiong K., Fan P., Zhang Y., Zhong Z. (2019). Power Minimization in SWIPT Networks with Coexisting Power-Splitting and Time-Switching Users Under Nonlinear EH Model. IEEE Internet Things J..

